# Strength–Ductility Balance of HIP+HT-Treated LPBF GH3536 Alloy via In Situ EBSD: The Role of Annealing Twins

**DOI:** 10.3390/ma18235306

**Published:** 2025-11-25

**Authors:** Changshuo Zhang, Xiaopeng Cheng, Junxia Lu, Shuai Huang, Bingqing Chen

**Affiliations:** 1 College of Materials Science and Engineering, Beijing University of Technology, Beijing 100124, China; 17861822597@163.com (C.Z.); xpcheng@bjut.edu.cn (X.C.); 2 AECC Beijing Institute of Aeronautical Materials, Beijing 100095, China; hshuai987@163.com (S.H.); hwtkjcbq1984@163.com (B.C.)

**Keywords:** GH3536 superalloy, laser powder bed fusion (LPBF), hot isostatic pressing (HIP), in situ EBSD tensile testing, plastic deformation, annealing twins

## Abstract

Nickel-based GH3536 alloys prepared by laser powder bed fusion (LPBF) exhibit a mismatch between strength and ductility during the tensile process, which severely restricts their engineering applications in the aerospace field. In order to optimize their performance, this study adopted hot isostatic pressing (HIP) and subsequent heat treatment (HT) to modify the material. The microstructural evolution of the HIP+HT-treated GH3536 alloy during deformation, including grain rotation, grain boundary migration, and dislocation slip transfer behaviors, was systematically investigated at room temperature using in situ tensile experiments. The relationship between the microstructure and mechanical properties was elucidated in greater depth by combining theoretical calculations. The experimental results show that after HIP+HT treatment, the elongation of the alloy increased significantly from 36.5% in the as-built LPBF condition to 45.3 ± 1.6% without a significant reduction in ultimate tensile strength. The plasticity enhancement is mainly attributed to the elimination of defects and the formation of annealing twins. In addition, the formation of substructures inside the grains also delays the fracture of the specimen to some extent. This study is expected to provide a reference for the subsequent optimization of the mechanical properties of alloys via heat treatment processes.

## 1. Introduction

As a typical solid-solution-strengthening nickel-based superalloy, GH3536 (the international brand name of Hastelloy X) exhibits good thermal stability, resistance to thermal oxidation, and excellent creep resistance [1]. It is widely applied in fields such as the aerospace, marine, nuclear reactor and chemical industries [2,3]. Compared with traditional forging technology, laser additive manufacturing technology represented by laser powder bed fusion (LPBF) has garnered significant attention due to the following advantages: it is not limited by the shape of the product parts, it has a short production cycle, and it has high material utilization and forming accuracy, providing an important approach for manufacturing complex parts with high geometric complexity, as well as for manufacturing high-performance GH3536 alloys [4].

However, during LPBF processing, the high cooling rates (10^7^ K/s) and high temperature gradients can lead to micro-compositional segregation, the formation of defects, and high residual stress in the fabricated alloys [5,6]. These microstructures can result in the distortion or cracking of parts, thereby affecting the mechanical properties of the material [7,8,9]. In the past several years, researchers have improved the performance of the alloy by modifying process parameters and via subsequent heat treatment. Although the plasticity of the alloy has been enhanced, it is difficult to achieve an optimal matching relationship between strength and ductility [10,11].

It has been demonstrated that the degree of strain localization in metallic materials exerts a pivotal influence on their mechanical properties, with reduced strain localization conferring superior mechanical properties [12]. Therefore, the key to obtaining an optimal strength–ductility synergy is to minimize strain localization and promote homogeneous plastic deformation. Based on this mechanism, several studies have been conducted. Liu et al. [13] introduced nano-precipitates as buffer zones and simultaneously promoted the stress release caused by the formation of stacking faults (SFs) at the two-phase interface, thereby reducing the stress localization at the two-phase interface. Meanwhile, this strategy also simulated the dislocation strengthening and precipitation strengthening effects, ultimately leading to a simultaneous increase in the YS and plasticity of the alloy by 96% and 66.7%, respectively. Kefu Gan et al. [14] mitigated strain localization and promoted uniform deformation of the material by introducing nano-twins, which resulted in a good balance between strength and plasticity. Ren et al. [15] combined in situ tensile tests and the crystal plasticity finite element method to study the relationship between the microstructural evolution and deformation behavior of alloys. The results showed that after heat treatment, the elongation of the material was improved by 80% without a significant reduction in tensile strength. This is mainly attributed to the selective obstruction of dislocations by annealing twins and the formation of serrated grain boundaries (GBs) after heat treatment. In general, although some scholars have studied the material strength–plasticity equilibrium through various methods to achieve a good balance between material strength and plasticity, the explanation from the microscopic point of view is insufficient, and there is still a lack of systematic research on the in situ deformation behavior of the material and on the relationship between its microstructure and mechanical properties.

Therefore, in this study, HIP and HT were used to improve the mechanical properties of the alloys and introduce annealing twins. The rotational deformation of grains, the evolution of grain boundaries, and the slip transfer of dislocations in the heat-treated GH3536 alloy during stretching were analyzed in detail using in situ EBSD. The interaction between the twin boundaries (TBs) and dislocations was analyzed by calculation, which further revealed the relationship between the microstructure and mechanical properties of the alloy.

## 2. Materials and Methods

### 2.1. Materials

**Figure 1:**
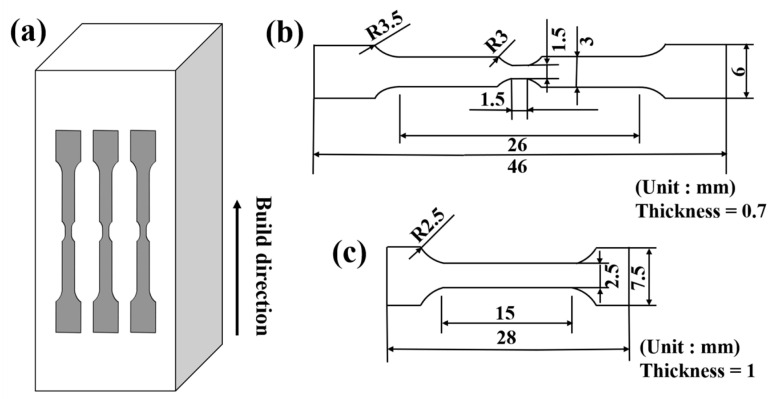
Preparation of specimens: (**a**) schematic of the specimen orientation; (**b**) geometries of the in situ tensile specimens; (**c**) geometries of the standard tensile specimens.

The LPBF GH3536 alloy used in this study was provided by Beijing Institute of Aeronautical Materials of Aero Engine Corporation of China and processed using an EOS M290 (EOS, Krailling, Germany) system equipped with a 400 W fiber laser. The heat treatment procedure was as follows: After the LPBF process, HIP post-treatment was conducted at 1195 °C and 170 MPa for 4 h, followed by furnace cooling to room temperature. Subsequently, the samples that had undergone HIP were subjected to further HT, specifically including homogenization at 1220 °C for 1 h, followed by furnace cooling to room temperature, solution treatment at 1130 °C for 0.5 h, and subsequent furnace cooling to room temperature. The GH3536 alloy was processed using electrical discharge machining (EDM), and the orientation of the tensile specimen was parallel to the forming direction, as shown in Figure 1a. Figure 1b illustrate the dimensions of the in situ tensile specimens, which were 46 mm in length, 6 mm in width, and 0.7 mm in thickness, as well as the area of the marked observation section: 1.5 × 1.5 mm^2^. Figure 1c shows the dimensions of the standard tensile specimens, which were 28 mm in length, 7.5 mm in width, and 1 mm in thickness.

### 2.2. Experimental Methods

The samples were ground in sequence using SiC sandpaper with a grit size of 240–5000 until the surface was smooth. Then, they were electrolytically polished using an electrolytic solution with a ratio of HClO_4_:C_2_H_5_OH = 9:1 at a polishing voltage of 18 V for 15 s. Subsequently, electrochemical etching was performed in a chemical mixture of H_2_C_2_O_4_:C_2_H_5_OH = 9:1 at an etching voltage of 7 V for 5 s, ensuring that the specimens were prepared for scanning electron microscope (SEM) high-magnification imaging and electron backscatter diffraction (EBSD) analysis.

To prepare the transmission sample, we first mechanically ground the sample thickness to approximately 70 μm using sandpaper. Subsequently, we cut it into a circular sample with a diameter of 3 mm using a punching machine and continued to grind it until the thickness was approximately 50 μm. Double-spray thinning of small disks was performed using an electrolyte composed of 10% perchloric acid and 90% ethanol at a constant voltage of 15 V and a constant temperature of −25 °C.

The microstructure of the materials was characterized by field emission SEM (TESCAN S8000, TESCAN, Brno, Czech Republic) and a transmission electron microscope (TEM, FEI Talos F200X, manufactured by FEI Company, Eindhoven, The Netherlands). The in situ tensile test was conducted using a self-developed in situ tensile system with a loading speed of 1 μm/s. This system can be paused at any time during the tensile process to capture the required SEM and EBSD images [16,17]. Tensile studies of titanium and nickel-based alloys at different temperatures have been conducted using this in situ testing system [9,18], and this can provide a more intuitive analysis of the relationship between the microstructure and mechanical properties.

## 3. Results

### 3.1. Microstructure Characterization

**Figure 2:**
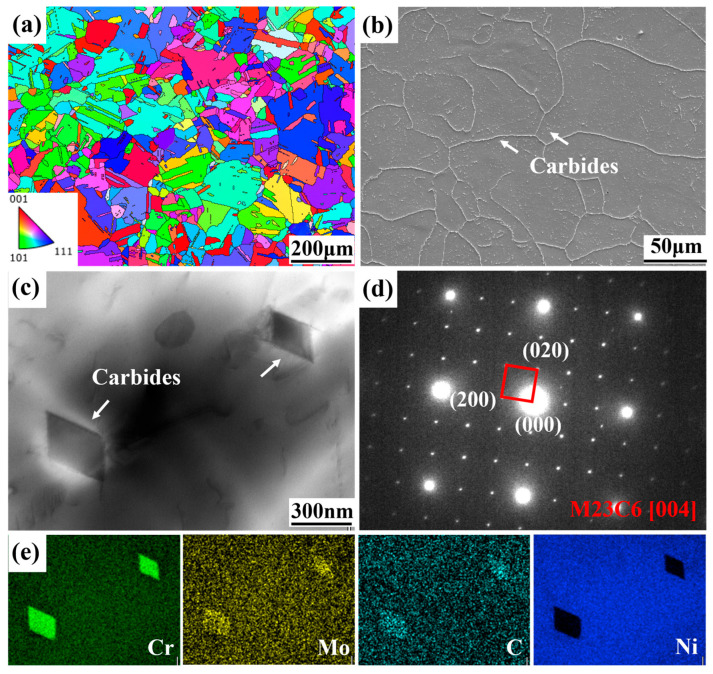
Microstructures of the HIP+HT LPBF GH3536 alloy: (**a**) IPF maps; (**b**) SEM images; (**c**,**d**) TEM pictures; (**e**) energy spectrum.

Figure 2a shows the EBSD inverse polar figure (IPF) of the HIP+HT-state LPBF GH3536 alloy. Compared with the as-built alloy reported in our previous study [15,19], after HIP+HT treatment in this study, the columnar crystals gradually transformed into equiaxed crystals, although a small amount of non-equiaxed grains could be found. This further confirms that recrystallization occurred during the heat treatment process of the alloy. The average grain size is 80.72 μm, which is greater than that of 26 μm for the as-built alloy [19]. Due to the fragmentation effect of the twin boundaries on the grains, the effective average grain size is 29.78 μm, which is close to the size of the as-built alloy. From the SEM picture in Figure 2b, almost no defects can be observed. Using Image Pro plus 6.0 software for statistical analysis on multiple regions, the defect density was about 0.07%, indicating a very compact structure. Compared with the as-built alloy, which has plenty of microcracks and holes, the results of our study suggest that HIP treatment can effectively reduce defects through the effects of high temperature and high pressure. Along the GBs and within the grains, a large number of bright precipitates could be found. By conducting electron diffraction analysis (Figure 2d) on the selected area and comparing it with the standard diffraction pattern, it was possible to determine that the phase has a face-centered cubic structure. The lattice constant can be obtained by measuring the interplanar spacing; that is, a = b = c = 1.06 nm. Combined with EDS mapping (Figure 2e), phases can be identified as Cr-rich M_23_C_6_ carbides with a grain size of approximately 300 nm. This is due to the Cr element having a high affinity for carbon, which can serve as a nucleation site for carbide precipitation. Additionally, the high-density dislocations in local areas in the as-built alloy also provide preferred locations for the nucleation of carbides [20].

### 3.2. Tensile Properties

Table 1 compares the yield strength (YS), ultimate tensile strength (UTS) and elongation to fracture of the GH3536 alloy at room temperature in different states. We found that, compared with the as-built specimen, the yield strength and tensile strength of LPBF GH3536 alloy were both decreased after HIP+HT treatment. The yield strength decreased from 562 MPa to 325 ± 15.6 MPa, and the ultimate tensile strength decreased from 785 MPa to 725 ± 12.8 MPa. Conversely, the elongation was improved, from 36.5% to 45.3 ± 1.6%. This decrease in strength is attributed to the increase in grain size and the decrease in residual stress, while the improvement in elongation is associated with the elimination of defects, the melt pool structure and the appearance of twins, as shown in Figure 2b. This is consistent with the conclusions in other studies [10,21]. In addition, compared with the wrought alloy, after HIP+HT treatment on the alloy under study in this research, the elongation essentially attained the usage standard, while the strength was slightly lower. This is related to the increase in grain size after treatment. However, the strength and elongation of the HIP+HT GH3536 alloy were both far higher than those of the cast counterpart.

### 3.3. In Situ Microstructural Evolution During Tensile Testing

**Figure 3:**
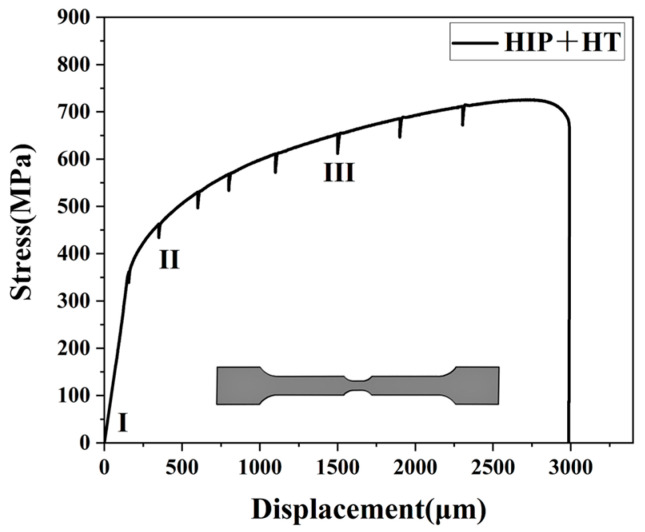
Stress–displacement curves obtained during the in situ tensile deformation of the HIP+HT LPBF GH3536 alloy.

To further study the relationship between the microstructure and mechanical properties, in situ EBSD tensile testing was carried out on the HIP+HT LPBF GH3536 alloy at room temperature. Figure 3 shows the stress–displacement curve established during the in situ tensile test. The stepped shape of the sample in the inset was designed to concentrate the deformation in the observation area during the in situ tensile test, so as to allow for observations of the evolution of the microstructure. The sawtooth shape in the curve is due to the stress relaxation caused by a pause created during stretching in the experiment to capture EBSD images, and then after loading, the stress returned to the original state.

**Figure 4:**
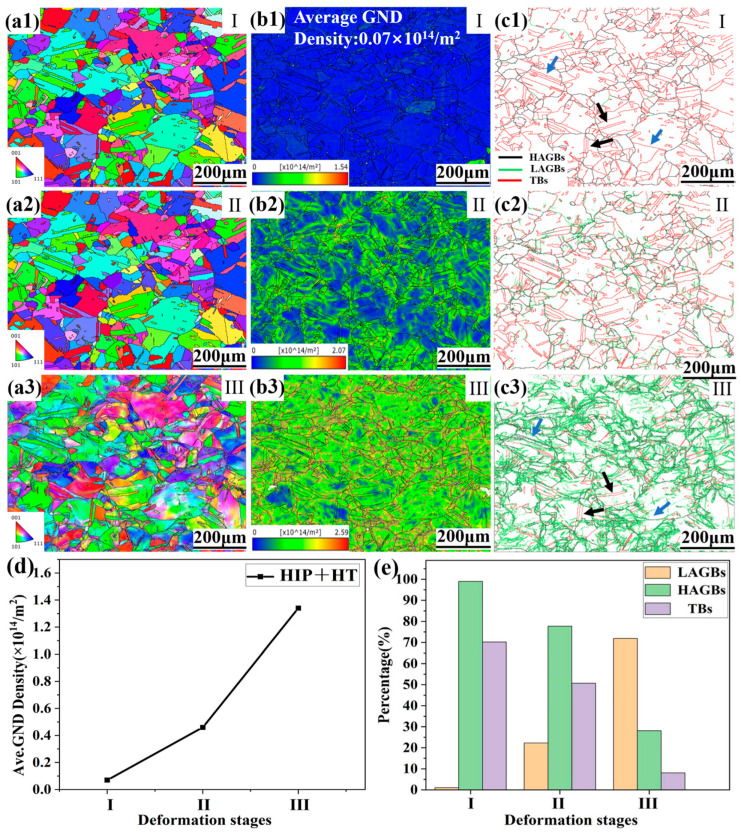
EBSD characterization of HIP+HT LPBF GH3536 alloy during in situ tensile testing: (**a1**–**a3**) in situ IPF variation; (**d**) and (**b1**–**b3**) GND changes; (**e**) and (**c1**–**c3**) GB changes.

Figure 4(a1–a3) show the in situ grain orientation distribution in the initial stage (I), the early plastic deformation (II) and the middle plastic deformation (III) shown in Figure 3. In the early stage, most of the grains exhibited a uniform orientation distribution. As the strain increased, sub-structures were formed on the surface. This effectively delayed the localization of strain, and thereby improved the plasticity of the specimen. Figure 4(b1–b3) correspond to the geometrically necessary dislocation (GND) in the three stages. It can be seen that the GND density in the initial stage was only 0.07 × 10^14^/m^2^, which is significantly lower than that of 0.8 × 10^14^/m^2^ of the as-built alloy [15]. This is due to the occurrence of recrystallization. The recrystallization process eliminates a large number of dislocations, which results in a decrease in residual stress. The change in color from blue to red in the box diagram in the left corner represents an increase in dislocation density. With the increase in deformation, the GND density gradually increases, as shown in Figure 4d, and stress concentration along the GBs occurs (Figure 4(b3)) due to dislocation accumulation. Figure 4(c1–c3) show the corresponding misorientation distributions of the GBs, where the black line represents the high-angle GBs (HAGBs, >15°), the green line represents the low-angle GBs (LAGBs, 2–15°), and the red line represents the twin GBs (TBs). Compared with the as-built LPBF GH3536 alloy [19], the treated alloy shows increased HAGBs and decreased LAGBs, as depicted in Figure 4(c1). This is due to the disappearance of sub-grain boundaries and the appearance of a high proportion of twins after heat treatment. In addition, the annealing twins formed through heat treatment not only divided the grains but also disrupted the spatial connectivity of random HAGBs. Therefore, excessive small grains can effectively prevent a decrease in strength and increase the tortuosity of the crack propagation path, which will delay the fracture of the specimen. Figure 4e shows the GB variation in the three stages. As the stretching proceeded, the proportion of HAGBs decreased from 99% to 28.1% and that of LAGBs increased from 1% to 71.9%. The nucleation position of the LAGBs is located in the lattice rotation regions in the IPF diagram and the stress concentration regions in the GND diagram. This indicates that the evolution of GBs is closely related to the movement of dislocations. As stretching proceeded, dislocations accumulated along the GBs and then were rearranged in the form of sub-grain boundaries, which induced an increase in LAGBs. In addition, the proportion of TBs gradually decreased with stretching, from the initial 70.2% to 8.1%. The interaction of dislocations and TBs disrupted the symmetrical relationship on both sides of the TBs, which caused the twins to transform into normal-angle GBs; see the blue arrows. The black arrows show the TBs that did not interact with the dislocations.

## 4. Discussion

**Figure 5:**
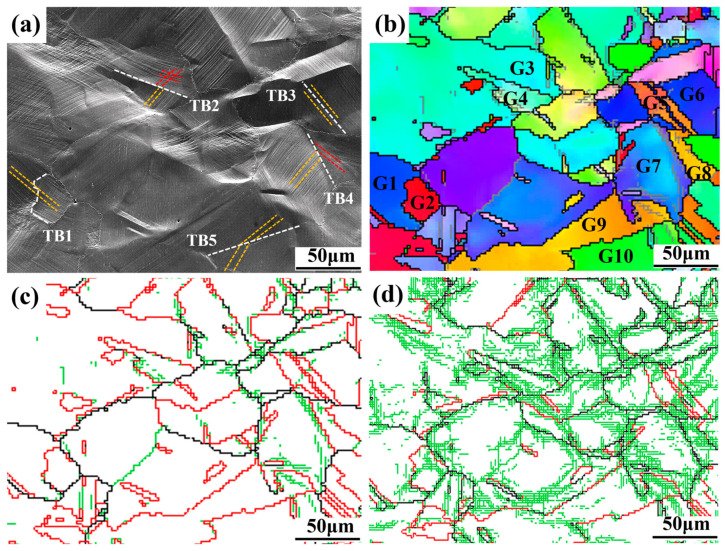
Interaction between TBs and dislocations during tensile process of HIP+HT LPBF GH3536 alloy: (**a**,**b**) SEM image and IPF diagram in stage II; (**c**,**d**) misorientation distribution of GBs in stage II and III.

From the above experimental results, it can be seen that the TBs had a great effect on plastic deformation. With deformation proceeding, some TBs transformed into normal-angle grain boundaries. To further explore this behavior, some grains and TB regions were selected for analysis, as shown in Figure 5. Figure 5a,b show SEM and IPF images of stage II in Figure 3, respectively, where the white dotted lines represent TBs, and the yellow and red lines both represent the slip lines. It can be seen that slip transfer occurred between grains 1 and 2, 5 and 6, while grains 3 and 4 showed no slip transfer during deformation. This indicates that the slip transfer capability is different from that of grains.

The geometric compatibility factor $m^{\prime }$ proposed by Luster and Morris [24] can quantify the ability for slip transfer between adjacent grains during dislocation slip:

(1) $$m^{\prime }=\;cos\kappa cos\psi$$

ψ represents the angle between the normal lines of the slip surface on both sides of the grain boundary, and κ represents the angle between the slip directions of adjacent grains. This method takes into account the coplanarity of the incoming and outgoing slip systems. The value of $m^{\prime }$ ranges from 0 to 1, where 0 indicates that the slip was hindered by the grain boundary, and 1 indicates that the grain boundary can be directly penetrated by the dislocation [25]. In general, $m^{\prime }$ > 0.7 denotes the occurrence of slip transfer [26]. Furthermore, when the Burgers vector remaining on the boundary is smaller, the possibility of slip transfer occurring is greater [27]. The magnitude ∆*b* of the residual Burgers vector after slip transfer can be calculated by the following formula [28]:

(2) $$∆b\;=\;\left|b\alpha \;-\;b\beta \right|=\;2\sin\left(\frac{\kappa }{2}\right)b,\;\kappa \;\leq \frac{\pi }{2}$$

From Figure 5a, five TBs were randomly selected. Geometric compatibility factors and residual Burgers vectors were calculated, and these are shown in Table 2. It can be seen that the $m^{\prime }$ values of TB1, TB3 and TB5 are 0.992, 0.9994, and 0.7814 (>0.7), respectively, and the value of ∆b is close to 0, which indicates that the slip lines were prone to transfer. Therefore, the accumulation of dislocations was prevented and the TBs were preserved, which in turn improved plasticity. The $m^{\prime }$ values of TB2 and TB4 are 0.2045 and 0.5097 (<0.7), respectively, and the value of ∆b is relatively large, indicating that it was not easy for the slip lines to pass through here. As stretching proceeded, the dislocations accumulated here, and the energy was relatively large, destroying the symmetric relationship between the two sides of the TB. By comparing the TB angle distribution diagrams of stage II (Figure 5c) and stage III (Figure 5d), it can be clearly observed that the TBs transformed into normal-angle GBs. The destruction of the TB structure effectively reduced the stress concentration and therefore delayed the fracture of the specimen. Overall, the appearance of TBs in GH3536 alloy can effectively alleviate the stress concentration, promote plastic deformation between grains, and achieve a good balance between the material’s strength and plasticity.

## 5. Conclusions

In this paper, the LPBF GH3536 alloy was treated via HIP+HT, and in situ EBSD stretching was carried out at room temperature using an in situ stretching system. The outcomes were then compared with those of LPBF state alloys. The interactions between the TBs and dislocations were calculated and analyzed. Thus, the following conclusions can be drawn.

1. After HIP+HT treatment, the surface defects of the material were basically eliminated (the defect density was only 0.07%). Under the effect of high temperature, the alloy underwent recrystallization. A large number of carbides precipitated at the grain boundaries, and within the grains, residual stress was released and a large number of annealing twins were generated.
2. For the annealing twins formed by HIP+HT, they were able to separate the grains, therefore effectively avoiding the strength reduction caused by excessive grain size.
3. During in situ stretching, the TBs selectively prevented the passage of dislocation. Some dislocations were able to pass through the TBs, avoiding the accumulation of dislocations at the TBs and therefore facilitating the improvement in the material’s plasticity. However, some dislocations were obstructed at the TBs and then accumulated here, causing the internal stress to increase and disrupting the symmetrical relationship between the TBs. This led to the transformation of the TBs into normal-angle GBs, effectively reducing stress concentrations and delaying the fracture of the sample.

## Figures and Tables

**Table 1:** Tensile properties of GH3536 alloy in different states.

State	YS (MPa)	UTS (MPa)	Elongation (%)	Ref.
HIP+HT	325 ± 15.6	725 ± 12.8	45.3 ± 1.6	This study
As-built	562	785	36.5	[19]
Cast	248	448	10	[22]
Wrought	376	783	46.5	[23]

**Table 2:** Summary of slip transfer geometric compatibility factor and residual Burgers vector.

TB	Grain	$m^{\prime }$	Δb
1	G1	0.992	0.0718
	G2		
2	G3	0.2045	0.9568
	G4		
3	G5	0.9994	0.02
	G6		
4	G7	0.5097	0.4474
	G8		
5	G9	0.7814	0.0065
	G10		

## Data Availability

The original contributions presented in this study are included in the article. Further inquiries can be directed to the corresponding author.
